# Supplementary material for: GSK2801 Reverses Paclitaxel Resistance in Anaplastic Thyroid Cancer Cell Lines through MYCN Downregulation
Source: Int J Mol Sci. 2023 Mar 22;24(6):5993. doi: 10.3390/ijms24065993 (PMC10054879; doi:10.3390/ijms24065993)
Supplement: Supplementary file 1 [file ijms-24-05993-s001.zip › ijms-2144412-supplementary.pdf]

	p-value vs control	
	8505C-PTX	SW1736-PTX
DMSO	-	-
0.5 µM	<0.01	ns
1 µM	<0.01	ns
2.5 µM	<0.0001	<0.05
5 µM	<0.01	<0.001
7.5 µM	ns	ns
10 µM	<0.01	<0.0001
15 µM	<0.0001	<0.0001
20 µM	<0.0001	<0.0001
30 µM	<0.0001	<0.0001

Supplementary Table S1. p-value of the MTT assay with VPC-70619. p-values obtained in the MTT assay with different doses of VPC-70619 alone compared with the control treatment are enlisted here.
